# Functional and structural characterization of *POR* splicing variants reveals pathogenic mechanisms in PORD

**DOI:** 10.3389/fendo.2026.1807069

**Published:** 2026-04-27

**Authors:** Xin Jie Zhang, Fei Yu Zhou, Xiao Wei Xu, Chao Wang, Ying Qian, Chao Yu Gong, Wen Chao Sheng, Jian Bo Shu, Chun Quan Cai, Ming Ying Zhang

**Affiliations:** 1 Children’s Hospital, Tianjin University/Tianjin Children’s Hospital, Tianjin, China; 2 Tianjin Pediatric Research Institute, Tianjin, China; 3 Tianjin Key Laboratory of Birth Defects for Prevention and Treatment, Tianjin, China; 4 Beijing Obstetrics and Gynecology Hospital, Capital Medical University, Beijing Maternal and Child Health Care Hospital, Beijing, China; 5 Graduate College of Tianjin Medical University, Tianjin, China; 6 Department of Endocrinology, Children’s Hospital, Tianjin University/Tianjin Children's Hospital, Tianjin, China

**Keywords:** genotype-phenotype correlation, minigene assay, P450 oxidoreductase deficiency, *POR*, splicing variant

## Abstract

**Background:**

Pathogenic *POR* variants cause P450 oxidoreductase deficiency, a rare steroidogenesis disorder. Missense changes are well characterized, but the clinical and molecular consequences of splicing defects remain unclear.

**Methods:**

We identified a novel homozygous splice variant (c.1249-2A>C) in a patient with disorders of sex development and Antley-Bixler syndrome -like skeletal malformations. By reviewing 12 published cases and performing minigene assays on five variants (c.731 + 1G>A, c.732-2A>T, c.947 + 1G>A, c.948-30G>A, c.1249-2A>C), we characterized their splicing outcomes. Structural consequences were predicted using AlphaFold; nonsense-mediated mRNA degradation was assessed for c.1249-2A>C using cycloheximide block.

**Results:**

c.731 + 1G>A and c.947 + 1G>A caused intron retention with premature termination, deleting FAD/NADPH-binding domains. c.732-2A>T and c.1249-2A>C skipped exons 8 and 12, which altered FAD-binding site conformation. c.1249-2A>C mRNA reduction was not rescued by cycloheximide, arguing against NMD and suggesting nuclear retention or intranuclear decay. We classified two variants as pathogenic (c.731 + 1G>A, c.947 + 1G>A), two as likely pathogenic (c.732-2A>T and the novel c.1249-2A>C), and one as likely benign (c.948-30G>A).

**Conclusion:**

Our findings establish that *POR* splicing variants, whether causing exon skipping or intron retention, disrupt essential domains and produce severe disease. Minigene-based functional testing enables precise variant classification and sharpens genotype-phenotype correlations, supporting improved diagnosis and informed genetic counseling.

## 1 Introduction

P450 oxidoreductase (POR) functions as the primary electron donor for microsomal cytochrome P450 enzymes (P450s) involved in steroidogenesis and drug metabolism (1). Human POR is composed of four conserved domains: an FMN-binding module, a connecting segment, an FAD-binding region, and an NADPH-binding site. These relay electrons sequentially from NADPH to FAD to FMN, finally delivering them to P450 enzymes (2, 3). The gene itself sits on chromosome 7q11.2 (OMIM#124015) and comprises 16 exons, with coding sequences starting from exon 2 (4). When POR is defective, the resulting phenotype is P450 oxidoreductase deficiency (PORD), a variant of congenital adrenal hyperplasia marked by cortisol insufficiency, disordered sex development, or skeletal abnormalities overlapping with Antley-Bixler syndrome (5). Beyond these classic features, the broader PORD phenotype includes absent or delayed puberty, menstrual disorders, infertility, maternal virilization during pregnancy, and ovarian macrocysts (6). Loss of POR activity directly reduces the function of multiple steroidogenic P450s in the adrenal cortex and gonads, including 21-hydroxylase, 17-hydroxylase and 17,20-lyase, disrupting adrenal and gonadal steroid synthesis (7). Variant-specific impairments in POR activity affect these enzymes to varying degrees, producing a phenotypic continuum from asymptomatic carriers to full Antley-Bixler syndrome (ABS)/disorders of sex development (DSD) presentations (8).

Although PORD has recognizable clinical features, definitive diagnosis still requires molecular analysis of the *POR* gene. Because the PORD phenotype overlaps with several other disorders, including ABS, polycystic ovary syndrome, and deficiencies of 21-hydroxylase or 17α-hydroxylase/17,20-lyase (9), accurate diagnosis demands careful clinical differentiation. In mild cases, subtle non-specific features are often overlooked until manifestations such as oligomenorrhea or infertility bring patients to medical attention (10), at which point they are frequently misdiagnosed as PCOS or functional oligomenorrhea (11). The contribution of *POR* variants to disease phenotype is more complex than previously appreciated. Compound heterozygosity has been identified in patients with both CAH and DSD (12), and *POR* polymorphisms can modify *CYP21A2* function, influencing both susceptibility to and severity of 21-hydroxylase deficiency (13). Case reports further document *POR* variants co-occurring with *SRY* and *DHX37* variants, together producing a combined phenotype of DSD and adrenal dysfunction (14). These findings demonstrate that *POR* variants can both cause disease independently and modulate other disease phenotypes. Thus, we recommend *POR* genetic testing for clinically suspected cases to prevent misdiagnosis and guide treatment.

Since PORD was first described in 2004 (15), the gnomAD database has cataloged 1,419 *POR* variants, including 55 splice-site alterations (16). Over 90 pathogenic variants are currently documented in ClinVar (https://www.ncbi.nlm.nih.gov/clinvar/?term=POR; accessed on 2024-01-15). Missense variants predominate and remain the most extensively studied (17, 18), whereas splicing variants are less common but frequently more deleterious. Unlike coding variants, splice-site alterations typically produce complex mRNA changes such as exon skipping or intron retention (19), making their pathological consequences particularly difficult to predict. Although splice-prediction tools efficiently flag most candidate spliceogenic variants, experimental validation is indispensable to confirm their effects and, together with genetic and clinical data, to achieve a definitive molecular diagnosis (20). To date, only scattered reports have experimentally assessed *POR* splicing defects, with limited follow-up mechanistic analysis. Consequently, many splicing variants remain classified as “Variants of Uncertain Significance” (VUS) due to the absence of functional validation, hindering precise genetic counseling (21, 22). We still lack a clear understanding of how particular *POR* splicing patterns correlate with clinical severity, which hampers accurate prognosis. Following the guidelines of the American College of Medical Genetics and Genomics and the Association for Molecular Pathology (ACMG/AMP) (23), minigene assays offer allele-specific, quantitative measurements of how exonic and intronic variants influence splicing. This functional assessment generates the evidence required to satisfy the ACMG PVS1/PS3 criterion, thereby enabling variant reclassification (24, 25).

Here, we describe a novel homozygous splicing variant (c.1249-2A>C) and comprehensively analyze five *POR* splicing variants using minigene assays. AlphaFold modeling was further employed to visualize and investigate the structural consequences of these variants. Our findings expand the mutational spectrum of *POR*, provide experimental evidence for variant classification, and refine genotype-phenotype correlations in PORD.

## 2 Methods

### 2.1 Summary of published data

We searched PubMed, Web of Science, HGMD, and ClinVar for genetic association studies and case reports using MeSH terms: “POR”, “P450 oxidoreductase”, “P450 oxidoreductase deficiency”, “Antley-Bixler syndrome”, “Disordered steroidogenesis”, and “Disorder of sex development”.

### 2.2 Clinical assessment

From our hospital’s CAH cohort, we identified one PORD patient who presented with micropenis at Tianjin Children’s Hospital. The diagnosis of PORD was considered based on clinical features suggestive of POR deficiency and was supported by endocrine abnormalities, imaging findings, and molecular analysis. No formal scoring system was used to define the PORD spectrum in this study; rather, inclusion was based on integrated clinical, biochemical, and genetic assessment. Clinical evaluation included history-taking, physical examination, laboratory testing, and imaging. Peripheral blood was drawn for genetic analysis at presentation. Parents provided written informed consent, and the study protocol was approved by the Ethics Committee of Tianjin Children’s Hospital (approval no.2025-LXKY-014).

### 2.3 Genetic analysis

Genomic DNA was extracted from peripheral blood using a commercial kit (Blood Genomic DNA Mini Kit) and quantified by NanoDrop 2000. Whole-exome sequencing (WES) was performed on the Illumina NovaSeq 6000 platform (MyGenostics). Bioinformatics analysis followed validated pipelines for variant calling. Variants were annotated using ClinVar, HGMD, gnomAD, dbSNP, and OMIM.

### 2.4 *In silico* analysis

Splicing impact of variants was computationally assessed using multiple *in silico* predictors, including NetGene2 (http://www.cbs.dtu.dk/services/NetGene2) (26), Berkeley Drosophila Genome Project (BDGP, https://www.fruitfly.org/seq_tools/splice.html) (27) and SpliceAI (http://asia.ensembl.org/Homo_sapiens/Tools/VEP or https://mobidetails.iurc.montp.inserm.fr/MD/) (28). The SpliceAI threshold for *de novo* or strengthened donor/acceptor delta score was set to >0.2. The three-dimensional structures of wild-type and mutant POR proteins were predicted with AlphaFold2 (https://colab.research.google.com/github/sokrypton/ColabFold/blob/main/AlphaFold2.ipynb) (29). The X-ray crystal structure of the human POR protein (PDB: 3QE2) was used as the wild-type template for the prediction. PyMOL Molecular Graphics System (Version 2.3, Schrödinger, LLC) was used for structural visualization and analysis of hydrogen bond interactions.

### 2.5 Minigene analysis

#### 2.5.1 Construction of minigene vectors

An *in vitro* minigene splicing assay was performed for the splicing variant using pSPL3 vectors which containing two tool exons (SD and SA). We designed primers containing *Eco*RI and *Bam*HI cleavage sites were used to amplify the target fragments including the flanking sequences of *POR* exons 7–10 and 11-12 (the reference transcript NM_001395413.1). Detailed of primers for vector construction was provided in **Supplementary Table 1** of **Supplementary Methods**. Two wild-type *POR* minigene vectors were constructed by cloning the polymerase chain reaction (PCR) products of exons 7–10 and exons 11–12 of the *POR* gene, amplified from human genomic DNA, into the pSPL3 expression vector using the ClonExpress Ultra One Step Cloning Kit (Vazyme Biotech Co., Ltd). The mutant-type minigene vector was constructed using the wild-type minigene vector as a template with the Fast Site-Directed Mutagenesis Kit (Tiangen Biotech (Beijing) Co., Ltd).

#### 2.5.2 Transfection of HEK293T and RT-PCR

Wild-type and mutant-type minigene vectors were separately transfected into Human Embryonic Kidney 293T (HEK293T) cell line using Lipofectamine^®^ 2000 (Thermo Fisher Scientific, Waltham, MA, USA). Total RNA was isolated after a 24-hour incubation period following transfection. Reverse transcription PCR (RT-PCR) was performed using the FastKing RT Kit (With gDNase) (Tiangen Biotech (Beijing) Co., Ltd). RT-PCR primers were SD: 5’-TCTGAGTCACCTGGACAACC-3’ and SA: 5’-ATCTCAGTGGTATTTGTGAGC-3’. The products were verified by 2.5% agarose gel electrophoresis and Sanger sequencing to evaluate the splicing effect.

#### 2.5.3 NMD sensitivity assay

NMD analysis was performed using a pcDNA3.1-based expression vector; detailed construction is described in **Supplementary Methods**. HEK293T cells were transfected with the wild-type and mutant constructs, and 20 μg/mL cycloheximide (CHX) was added for 6-hours at 36–48 hours post-transfection to inhibit protein synthesis and stabilize potential NMD-targeted mRNAs. Total RNA and protein were subsequently extracted for qPCR and Western blot analysis, respectively.

#### 2.5.4 Western blot quantification

Protein extracts were separated by SDS-PAGE and transferred to PVDF membranes. Membranes were probed with rabbit anti-POR antibody (1:10000, Abcam, ab180597) and mouse anti-GAPDH (1:5000, Abcam, ab181602). Bands were visualized by enhanced chemiluminescence and captured using a ChemiDoc Touch system (Bio-Rad). Intensities were quantified with Image Lab software and normalized to GAPDH. Relative POR expression was calculated as the ratio to wild-type (set as 1.0).

#### 2.5.5 Statistical analysis

Data represent mean ± SD from three independent experiments. Group comparisons used one-way ANOVA with Tukey’s *post-hoc* test. *P<0.05, **P<0.01, ***P<0.001, ****P<0.0001.

### 2.6 ACMG/AMP variant classification

We classified variants according to ACMG/AMP guidelines (23). Evidence codes included: PS3 (functional data supporting pathogenicity), BS3 (functional data supporting benignity), PM2 (absence in population databases), PVS1 (null variant in known mechanism), and PM3/PP1 (allelic configuration and segregation). Final classifications followed standard ACMG point thresholds (30).

## 3 Results

### 3.1 Clinical and genetic characteristics of 13 PORD patients with *POR* splice variants

**Figure 1:**
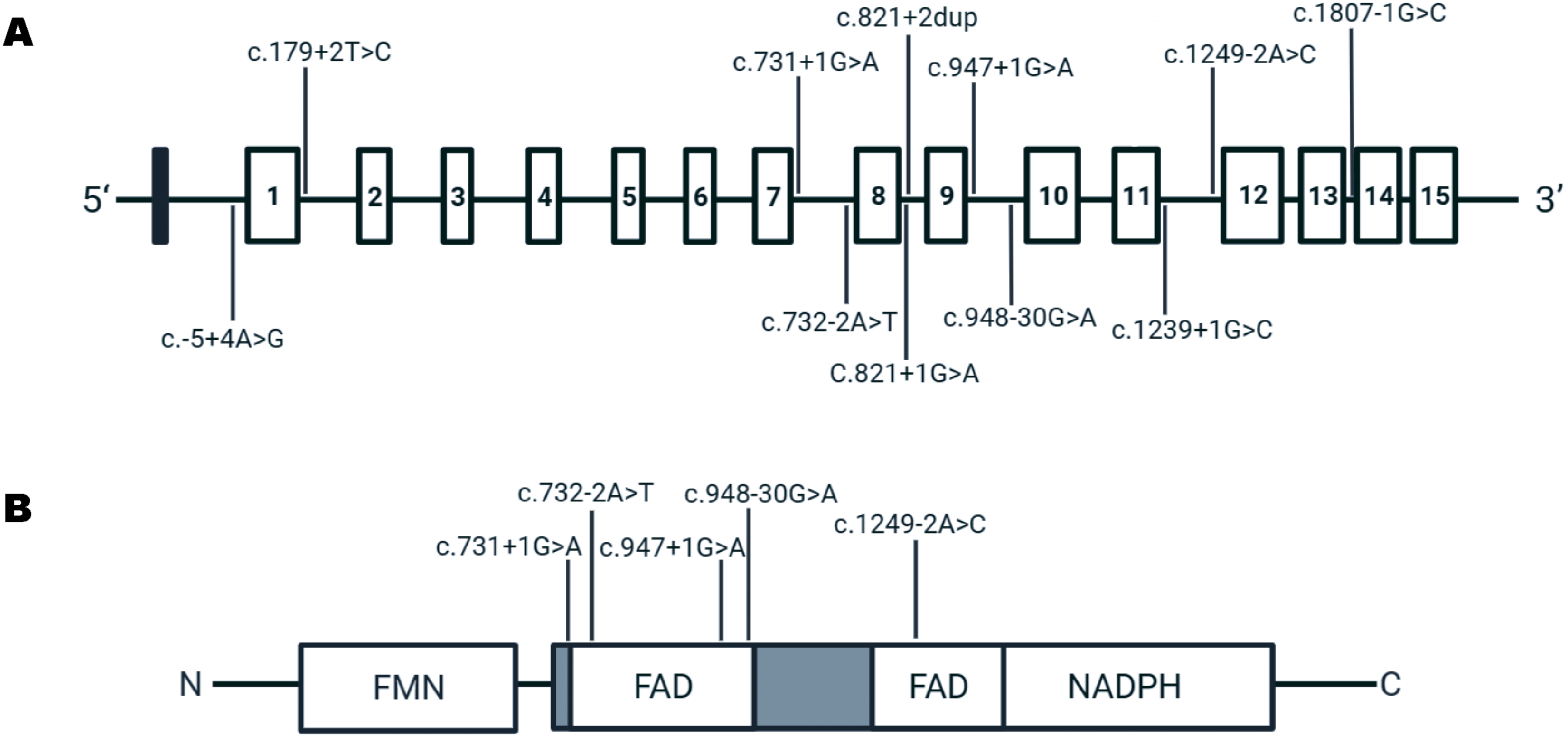
Schematic of the *POR* gene and localization of splice-site variants. **(A)** Exon-intron structure of the *POR* gene. Coding exons are shown as open boxes; untranslated regions (UTRs) are filled black boxes. Arrows indicate the locations of variants investigated in this study. **(B)** Linear schematic of POR protein domains showing variant positions relative to the FMN-, FAD-, and NADPH-binding domains.

Through systematic literature review and clinical screening, we identified a total of 13 patients with *POR* splice variants, including 12 previously reported cases and one novel case from our institution (**Table 1**; **Figure 1**). Among these patients, six (6/13) carried splice variants in compound heterozygosity with the missense variant A287P. No significant gender difference was observed. The majority of patients (9/13) presented with skeletal abnormalities. Except for patients 5 and 6, who were siblings, all others were unrelated.

**Table 1:** Genotypes, clinical manifestations, and in silico predictions of *POR* splice-site variants.

Patient	Sex	Ethnicity	Mutant allele 1	Mutant allele 2	Clinical significance	Authors, year	NetGene2	BDGP	SpliceAI
1	M (46, XY)	China	c.1249-2A>C	c.1249-2A>C	1. DSD (micropenis)	This study	Loss of acceptor splice site	N/A	Loss of acceptor splice site (AL = 1.00)
					2. ABS				
2	F (46, XX)	Turkish	c.-5 + 4A>G	p.L374H	1.DSD (virilization of external genitalia at birth, posterior labial fusion, rugged major labia, no palpable gonad and clitoromegaly)	Shaheena Parween et al., 2016 (31)	N/A	Loss of donor splice site	Gain of donor splice site (DG = 0.44)
					2. Maternal virilization				
3	F (46, XX)	Japanese	c.731 + 1G>A	p.R457H	1. ABS (craniosynostosis, hypertelorism, mild choanal narrowing, radiohumeral synostosis, mid-face hypoplasia, arachnodactyly, rocker-bottom feet)	Christa E Flück et al., 2004 (15)	Loss of donor splice site	Loss of donor splice site	Loss of donor splice site (DL = 1.00)
					2. DSD (clitoromegaly, labial fusion and a single uro-genital orifice)				
					3. Maternal virilization				
					4. Adrenal insufficiency (mild hyponatremia, hyperkalemia and hypoglycemia)				
4	–	British	c.732-2A>T	p.R457H	1. ABS (Craniosynostosis Brachycephaly, RH/Rua Synostosis, Femoral Bowing)	Kumar et al.,1997 (32)	Loss of acceptor splice site	N/A	Loss of acceptor splice site (AL = 1.00)
					2. DSD (a urogenital sinus, vestigial uterus, and a posteriorly placed septated vagina)				
5	F (46, XX)	USA	c.732-2A>T	p.A287P	1. ABS (Midface hypoplasia Craniosynostosis, Phalangeal malformations, Large joint synostosis, Femoral bowing, Anteriorly placed anus, single umbilical artery, dysplastic ears)	Nils Krone et al.,2012 (33).			
					2. Abnormal Steroids				
6	M (46, XY)	USA	c.732-2A>T	p.A287P	1. ABS (Midface hypoplasia Craniosynostosis, Phalangeal malformations, Large joint synostosis, Femoral bowing, Dysplastic ears)				
					2. Abnormal Steroids				
					3. DSD (micropenis and perineoscrotal hypospadias)				
7	–	French (fetus)	c.732-2A>T	p.A287P	1. ABS (trapezoidocephaly, severe midface hypoplasia, depressed nasal bridge, short philtrum, hypoplasia of the maxillary and nasal bones, and dysplastic low-set ears)	Elena Oldani et al.,2015 (34)			
					2. DSD (hypoplasia of the labia majora, with small urinary meatus, vaginal hypoplasia, clitoromegaly, and bicornuate uterus)				
8	M (46, XY)	UK	c.821 + 2dupT	p.Q455RfsX544	1.ABS (Midface hypoplasia Craniosynostosis, Phalangeal malformations, Large joint synostosis, Femoral bowing, Arnold-Chiari malformation)	Nils Krone et al.,2012 (33)	Loss of donor splice site	Loss of donor splice site	Loss of donor splice site (DL = 0.64)
					2. Abnormal Steroids				
					3. DSD				
9	M (46, XY)	UK	c.821 + 2dupT	p.A287P	1.ABS (Midface Hypoplasia, Craniosynostosis, Large joint synostosis)				
					2. Abnormal Steroids				
10	–	British	c.821 + 2dupT	p.A287P	1. ABS (Craniosynostosis Brachycephaly, Midface Hypoplasia, Proptosis)	Ningwu Huang et al.,2005 (35)			
					2. Abnormal Steroids				
11	F (46, XX)	UK	c.947 + 1G>A	p.A287P	1. DSD	Nils Krone et al.,2012 (33)	Loss of donor splice site	Loss of donor and acceptor splice site	Loss of donor splice site (DL = 0.97)
					2. Abnormal Steroids				
					3. ABS (Midface hypoplasia, Craniosynostosis, Phalangeal malformations, Large joint synostosis)				
12	XY?	NN	c.948-30G>A	normal	Undiagnosed	L A Hughes et al., 2019 (36)	N/A	N/A	N/A (AG = 0.04, AL = 0.00, DG = 0.00, DL = 0.00)
13	F (46, XX)	China	c.1807-1G>C	p.399_401delPSE	1. DSD (aberrant genitalia, stenosis of the vagina, and multiple ovarian cysts)	Tao Zhang et al., 2020 (37)	Loss of acceptor splice site	Loss of acceptor splice site	Loss of acceptor splice site (AL = 0.94)
					2. Abnormal Steroids				

“NA” indicates that the prediction shows no splicing alteration. “AG/AL” indicates acceptor gain/loss and “DG/DL” indicates donor gain/loss.

The proband from our institution (patient 1 in **Table 1**) was a 6-month-old boy who was admitted to the hospital primarily for micropenis. Born to healthy, non-consanguineous parents, prenatal ultrasound at 30 weeks of gestation had identified microfoot malformations. At presentation, the proband measured 74 cm in height (+2 to +3 SD). Examination revealed orbital hypertelorism, prominent ears, and bilateral hand contractures: he could not flex his thumbs, index fingers, or little fingers. Foot radiographs showed short fourth and fifth metatarsals. Examination showed micropenis (1.5 cm) and bilateral 1 ml testes. Whole-exome sequencing identified a homozygous splice-site variant c.1249-2A>C (NM_001395413.1) in *POR*. We confirmed parental carrier status by Sanger sequencing. No reports of this variant exist in the literature or ClinVar.

### 3.2 *In silico* splicing variant pathogenicity prediction

This study analyzed eight splicing variants in the *POR* gene using three splicing prediction tools: NetGene2, BDGP, and SpliceAI. Results showed that variants c.731 + 1G>A, c.821 + 2dupT and c.1807-1G>C were consistently predicted by all three tools to cause loss-of-function splicing; Variant c.948-30G>A was predicted by all three tools to have no effect on splicing; while variant c.947 + 1G>A was predicted by all three tools to result in loss of the 5’ donor splice site, with BDGP additionally predicting potential impact on the 3’ acceptor splice site. Among the remaining three variants, at least one prediction tool indicated potential disruption of splicing site function. Detailed results are shown in **Table 1**.

We selected five representative variants (c.731 + 1G>A, c.732-2A>T, c.947 + 1G>A, c.948-30G>A and c.1249-2A>C) for minigene validation, covering canonical disruption, exon skipping, and predicted benign categories. c.1807-1G>C and three additional discordant variants were not experimentally assessed.

### 3.3 Functional characterization by minigene assay

**Figure 2:**
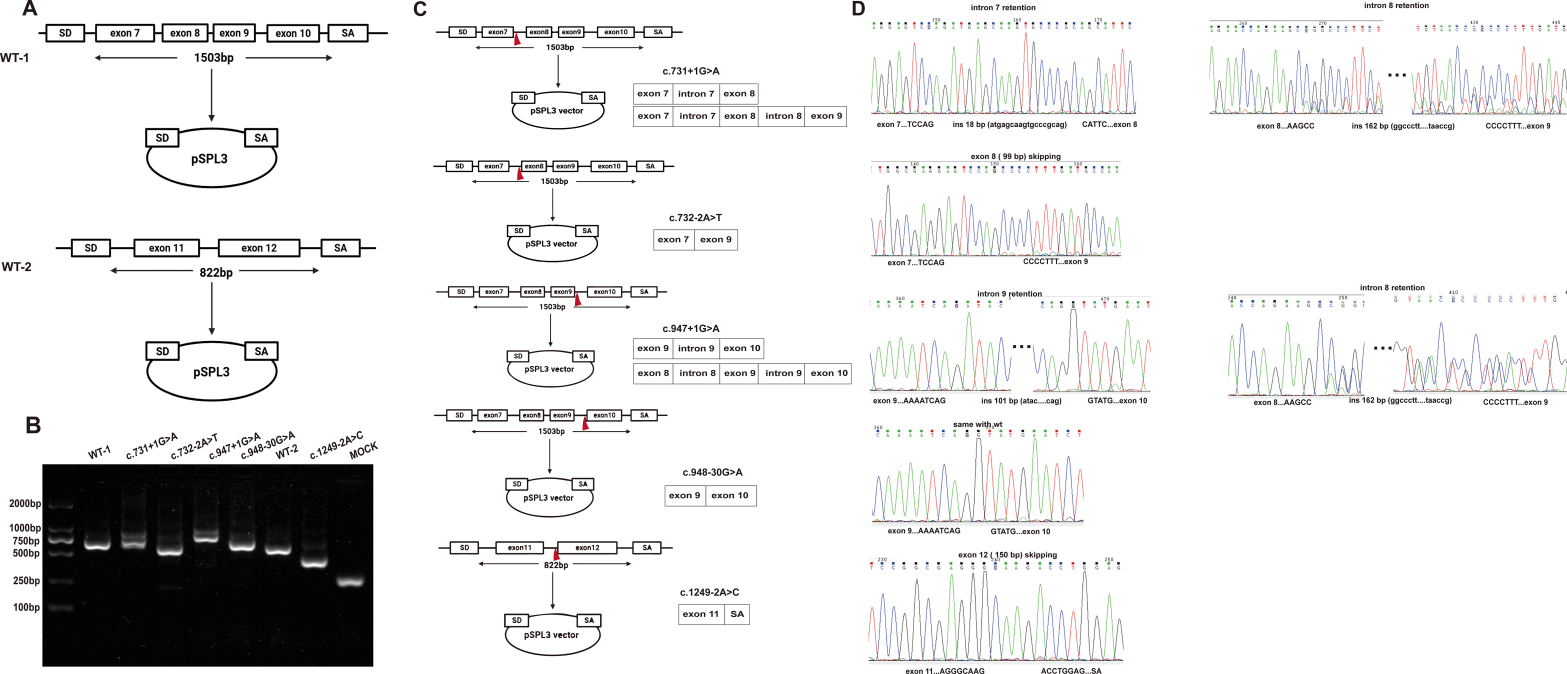
Functional characterization of *POR* splice-site variants by minigene assay. **(A)** Schematic of pSPL3 minigene constructs. Genomic fragments containing target exons and flanking intronic sequences were inserted between the SD and SA exons. **(B)** Agarose gel electrophoresis of RT-PCR products from HEK293T cells transfected with wild-type or mutant minigenes. **(C)** A schematic diagram showing the location of the variants in the minigene expression constructs (indicated by the red triangles) and the results of abnormal splicing. **(D)** Sanger sequencing validation.

Because of the instability of *in silico* analyses, we constructed two minigene expression vectors to investigate the pathogenic mechanisms of splice variants (**Figure 2A**). Gel electrophoresis (**Figure 2B**) results showed that both c.731 + 1G>A and c.947 + 1G>A mutant samples produced two longer cDNA products than the wild-type. Abnormal splicing patterns are schematically illustrated (**Figure 2C**). Direct sequencing of PCR products confirmed an 18 bp (from intron 7) insertion in the c.731 + 1G>A mutant cDNA. Additionally, this variant exhibited a compound insertion of 162 bp and 18 bp. Sanger sequencing revealed that the c.947 + 1G>A mutation resulted in two splicing products: one retaining intron 9 (total 101 bp), and another retaining intron 9 with an additional 162 bp sequence insertion. This 162 bp sequence matches the insertion detected in c.731 + 1G>A, both originating from intron 8. Conversely, cDNA bands from c.732-2A>T and c.1249-2A>C samples exhibited shorter lengths compared to the wild-type. Exon skipping occurred in exon 8 of the c.732-2A>T sample and in exon 12 of the c.1249-2A>C sample. The cDNA product length of the c.948-30G>A sample was consistent with the wild-type. All of the above results were confirmed by Sanger sequencing (**Figure 2D**).

### 3.4 Decreased mRNA expression of c.1249-2A>C via NMD

**Figure 3:**
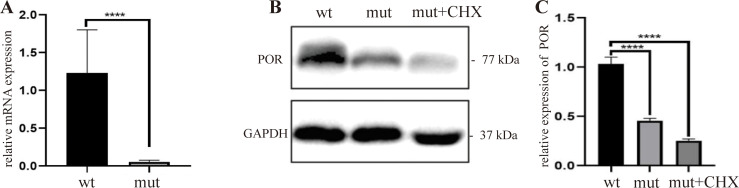
Effect of CHX on *POR* expression in HEK293T cells. **(A)** Relative *POR* mRNA levels by qPCR. **(B)** Western blot of POR and GAPDH. **(C)** Quantified POR protein levels (relative expression, ratio to wild-type set as 1. ****P < 0.0001.

Variant c.1249-2A>C reduced mRNA to 4.6% of wild-type levels (**Figure 3A**), with matching protein reduction (**Figure 3B**). We treated cells with cycloheximide (CHX) to test NMD involvement. Unexpectedly, CHX failed to rescue either mRNA or protein; protein levels actually dropped further with CHX treatment (**Figure 3C**). This argues against NMD and points to alternative degradation mechanisms.

### 3.5 Structural modeling of splice variants

**Figure 4:**
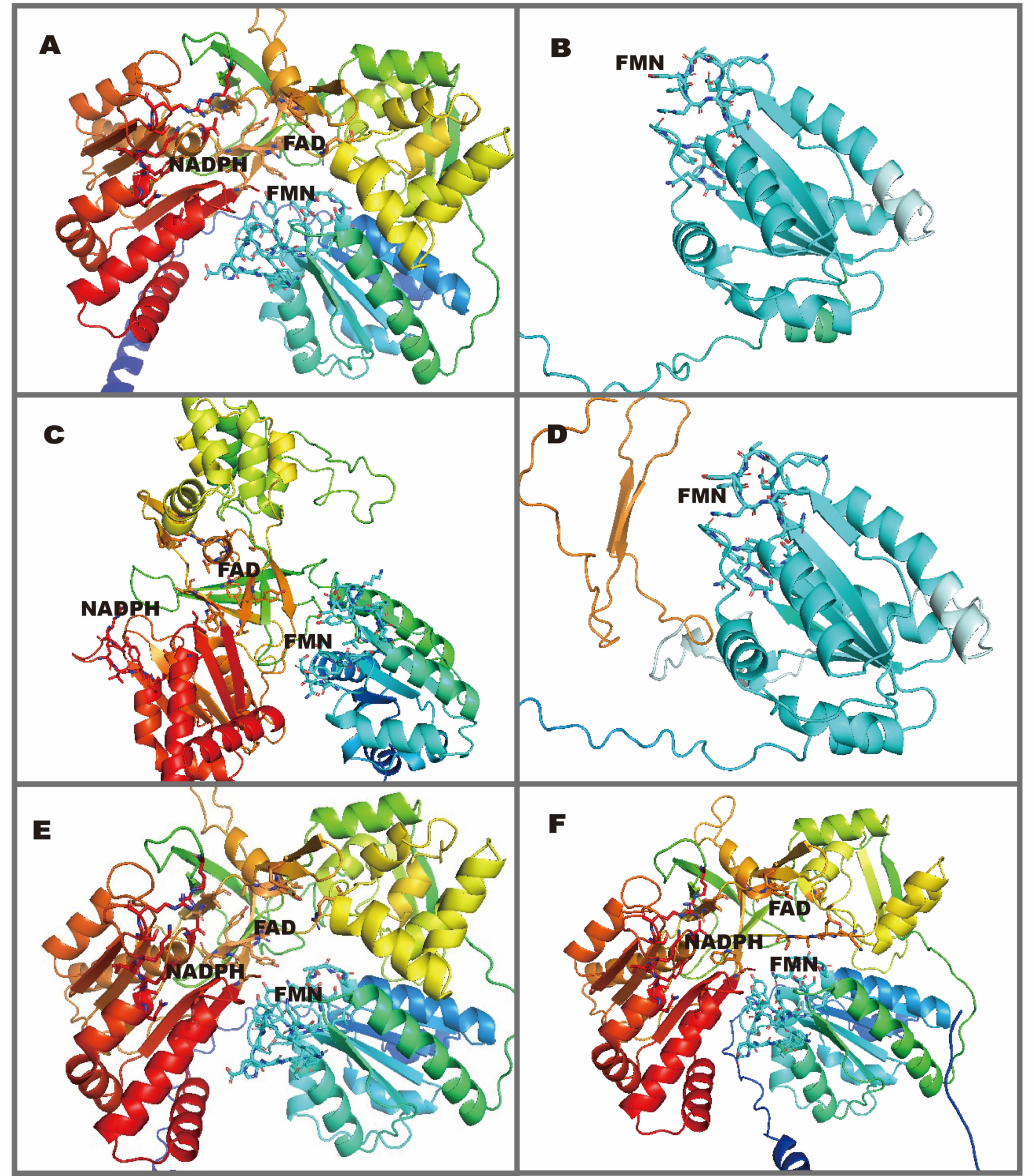
AlphaFold2-predicted structures of wild-type and mutant POR proteins. **(A)** Wild-type POR structure showing functional domains: NADPH-binding (red), FAD-binding (yellow), and FMN-binding (blue). **(B-F)** Mutant structures: **(B)** c.731 + 1G>A (truncated); **(C)** c.732-2A>T (exon 8 skipping); **(D)** c.947 + 1G>A (truncated); **(E)** c.948-30G>A (no structural change); **(F)** c.1249-2A>C (exon 12 skipping).

AlphaFold-predicted structures revealed distinct consequences of splicing disruption (**Figure 4**). The c.948-30G>A variant yielded a structure indistinguishable from wild-type POR. However, c.731 + 1G>A and c.947 + 1G>A, which cause frameshifts and premature truncation, showed complete loss of the FAD and NADPH domains, retaining only the FMN domain. Variants causing internal exon skipping (c.732-2A>T and c.1249-2A>C) exhibited more subtle conformational changes, with preserved domain architecture.

### 3.6 ACMG pathogenicity classification

We classified five POR splice variants using ACMG criteria (**Table 2**). Discordance among *in silico* tools precluded PP3 evidence. Experimental validation supported: PVS1 for c.731 + 1G>A, PVS1-strong for c.947 + 1G>A, PS3 for c.732-2A>T and c.1249-2A>C, and BS3 for c.948-30G>A. Integrating functional, population (PM2), splice-site position, and allelic data, we designated four variants as pathogenic or likely pathogenic and c.948-30G>A as likely benign. The novel c.1249-2A>C variant, which showed exon 12 skipping without clear loss of function, was classified as likely pathogenic based on PS3 and PM2.

**Table 2:** Pathogenicity assessment of *POR* splice-site variants by ACMG/AMP criteria.

Variant	PS3/BS3 (function)	BA1/BS1/BS2/PM2 (frequency codes)	PP4 (phenotypic specificity)	PS4 (case-control data/rare variant)	PP1/BS4 (cosegregation)	PS2/PM6 (denovo occurrence)	PM3/BP2 (allelic data)	BP5 (alternate molecular basis for disease)	PVS1	Re-evaluated classification
c.731 + 1G>A	–	PM2	–	–	–	–	PM3	–	PVS1	P (11)
c.732-2A>T	PS3	PM2	–	–	PP1	–	PM3	–	–	LP (8)
c.947 + 1G>A	–	PM2	–	–	–	–	PM3	–	PVS1-strong	P (11)
c.948-30G>A	PS3	PM2	–	–	–	–	–	–	–	LB (-1)
c.1249-2A>C	PS3	PM2	–	–	–	–	–	–	–	LP (5)

P, pathogenic; LP, likely pathogenic; VUS, variant of uncertain significance; LB, likely benign; B, benign.

## 4 Discussion

In this study, we determined the pathogenicity of a novel homozygous splice variant (c.1249-2A>C) in a patient with PORD and re-analyzed four previously reported splice-site variants. Minigene assays bridged the gap between *in silico* scores and measurable splicing outcomes, allowing unambiguous classification of all five variants and linking computational prediction to clinical diagnosis.

Minigene analysis revealed several distinct splicing alterations. Both c.731 + 1G>A and c.947 + 1G>A caused intron retention, generating multiple transcripts with premature termination codons (PTCs). AlphaFold2 modeling shows these truncations remove the entire C-terminal FAD and NADPH binding domains, which are essential for electron transfer to cytochrome P450 enzymes (1), predicting complete loss of catalytic activity. In the case of c.731 + 1G>A, our experimental data refine a previous report of intron retention (15); the affected individual presented with classic PORD features, including skeletal malformations, DSD, and abnormal steroid profiles. According to a standardized scoring system for skeletal anomalies (33), the patient carrying the c.947 + 1G>A variant presented with severe malformations including midface hypoplasia, craniosynostosis, and hand and foot malformations. These truncations correlate with the severe clinical phenotypes observed. In contrast, c.948-30G>A (detected heterozygously in a patient with ambiguous genitalia (36)) produced a wild-type splicing pattern and is therefore reclassified as likely benign. The original DSD panel used for this patient covers only 30 genes and lacks CNV detection, suggesting that the patient may harbor other pathogenic variants that were not identified.

The novel variant c.1249-2A>C leads to the skipping of exon 12. Although this does not immediately result in protein truncation, AlphaFold prediction model suggests that deletion of this specific region modifies the structure of the protein’s FAD-binding site. This structural alteration may weaken cofactor binding and consequently destabilizes the enzyme, reducing or abolishing its catalytic activity (38). Importantly, preservation of the reading frame does not necessarily imply a mild or benign phenotype. In-frame exon skipping may still disrupt critical structural elements or functional domains and therefore remain clinically significant. In *POR*, even relatively small in-frame alterations may compromise regions involved in FAD/NADPH binding or electron transfer, thereby substantially reducing enzymatic activity. CHX treatment failed to rescue the mRNA decline, suggesting nuclear retention (39) or intranuclear decay (40) rather than NMD as the primary mechanism. We cannot exclude a minor NMD component because of CHX dosage and cytotoxicity (41) constraints, but the bulk of evidence argues against NMD as the driving pathogenic mechanism. Complete loss of POR function is embryonic lethal, as demonstrated by germline knockout mouse models (42). Consistent with this, no patients carrying biallelic nonsense variants have been reported to date (37). These findings suggest that c.1249-2A>C is a hypomorphic allele (retaining residual activity) rather than a complete loss of function (LoF) allele. Taken together, these findings suggest that c.1249-2A>C is unlikely to represent a complete loss-of-function allele, although residual activity remains to be formally demonstrated.

Minigene and clinical data tighten the genotype-phenotype link in PORD. Specifically, c.732-2A>T causes exon 8 skipping and consistently associates with severe skeletal and DSD features across four reported patients (**Table 1**). All were compound heterozygotes carrying c.732-2A>T in trans with hypomorphic alleles (A287P (33, 34) or R457H (35)), and all exhibited severe phenotypes. The severity of skeletal malformations in PORD hinges on the specific variant combination (43). Patients with two A287P alleles show only mild disease (3), and even R457H homozygotes fare better than those carrying one null allele (4). This positions c.732-2A>T as a null allele: when paired with hypomorphic partners, the resulting functional hemizygosity (or insufficient total POR activity) likely accounts for the severe skeletal and DSD features observed. Although some in-frame splicing variants may retain partial residual function and therefore be associated with milder phenotypes, the clinical impact of a given variant ultimately depends on whether the resulting transcript disrupts essential structural elements or functional domains. These results support the high pathogenicity of this splice defect and demonstrate that even within the complex PORD spectrum, splice-site variants can be used to estimate physical severity and guide clinical management.

Accurate interpretation of splicing variants remains a major challenge in genetic counseling. The most recent guidelines from the ClinGen SVI Splicing Subgroup recommend a robust framework for determining the pathogenicity of splicing variants, which involves assigning varying levels of functional evidence based on *in silico* predictions and experimental data (23). Using minigene assays, we classified five *POR* variants (two pathogenic, two likely pathogenic and one likely benign; **Table 2**). Disruption at the canonical ±1/± 2 positions is usually predictable (44), yet our data underscore the value of functional testing. While *in silico* tools gave conflicting scores for c.732-2A>T and c.1249-2A>C, they correctly predicted high-impact disruption for c.731 + 1G>A and c.947 + 1G>A. Nevertheless, only experimental validation revealed the exact nature of the splicing defects, including the specific aberrant transcripts generated. The minigene vectors generated here now provide a ready platform for validating additional VUS splice-site variants in *POR*. In addition, when interpreting the clinical significance of variants, transcript-level alterations should be considered together with their predicted functional consequences, protein domain features, and the patient phenotype.

This study has several limitations. First, although the minigene assay reliably recapitulates splicing (45), its patterns may not accurately reflect the *in vivo* situation. Second, we modeled the variant protein on the assumption that the variant does not trigger NMD. While we have ruled out NMD for c.1249-2A>C, the remaining variants still need formal verification. Previous studies have demonstrated that the A287P isoform maintains full enzymatic activity but causes disease through reduced stability rather than catalytic defects (46). Therefore, more functional tests are required for the variants found here in order to ascertain whether pathogenicity stems from degradation-induced low protein levels or catalytic defects. While the relative contributions of transcriptional decay versus protein instability require further investigation, the combination of aberrant splicing and functional impairment fulfills ACMG criteria for pathogenicity (PVS1/PS3), supporting clinical relevance. Given the substantial phenotypic heterogeneity of PORD, larger cohorts and additional biochemical studies will be necessary to further refine genotype–phenotype correlations, especially for splice variants with potentially distinct functional consequences. In particular, the limited number of reported cases and the lack of direct protein-level evidence currently constrain our ability to determine how specific splice alterations influence clinical severity.

In summary, we identified a patient carrying the novel homozygous c.1249-2A>C splice-site variant and systematically analyzed five previously reported *POR* splicing defects. Minigene assays demonstrated aberrant splicing for four variants (c.731 + 1G>A, c.732-2A>T, c.947 + 1G>A, and c.1249-2A>C) and confirmed the benign nature of c.948-30G>A. We further delineated a genotype–phenotype correlation between splicing error type and clinical severity. Although limited to ex vivo analysis, our findings underscore the value of minigene splicing assays for accurate PORD diagnosis and provide a validated platform for assessing future variants of uncertain significance.

## Data Availability

The original contributions presented in the study are included in the article/**Supplementary Material**. Further inquiries can be directed to the corresponding authors.
