# Supplementary material for: Functional and structural characterization of POR splicing variants reveals pathogenic mechanisms in PORD
Source: Front Endocrinol (Lausanne). 2026 Apr 27;17:1807069. doi: 10.3389/fendo.2026.1807069 (PMC13158119; doi:10.3389/fendo.2026.1807069)
Supplement: Supplementary file 1 [file DataSheet1.doc]

**1 Supplementary Data**

**Construction of Full-Length POR Expression Vectors for NMD Assay**

To assess the impact of the splice variant c.1249-2A>C on mRNA stability and potential nonsense-mediated mRNA decay (NMD), we constructed a full-length *POR* expression vector using the pcDNA3.1 backbone. Given that the variant is located at the 3′ splice acceptor site of exon 12, intronic sequences flanking this exon were retained to preserve the natural splice junction context for accurate evaluation of splicing defects.

The *POR* gene fragment was amplified in three overlapping fragments (P1, P2, and P3) as illustrated in Figure S1, with primers listed in Table S2. These fragments were cloned sequentially into the pcDNA3.1 vector using restriction enzyme sites (*Bam*HI and *Eco*RI) incorporated into the primers. The final construct thus contains the complete coding sequence of POR, with the native intronic sequences flanking exon 12 preserved. Using the Fast Site-directed Mutagenesis Kit, mutant plasmid was obtained through primer-directed mutagenesis. All plasmids were verified by Sanger sequencing before transfection.

**2 Supplementary Figures and Tables**

Table S1. Primers for Vector Construction in Minigene assay

| Explanation | Primers | Sequence (5′ →3′) |
| --- | --- | --- |
| Amplicon 7 | P1 | ggtacgggatcaccagaattcTGTCGCTGGGTGCCCCAG |
|  | P2 | tgctgcagatACCAAGGTCCCTCCTCGACC |
| Amplicon 8 | P3 | ggaccttggtATCTGCAGCAGGGGCTCC |
|  | P4 | tgcagttctAGTAAGGTGGCTAAGTGAGCTCAGT |
| Amplicon 9/10 | P5 | gccaccttactAGAACTGCATTGGACCAGGCT |
|  | P6 | atcaccagatatctgggatccCCCCTAGGGCCACCCCGC |
| Amplicon 11 | P7 | ggtacgggatcaccagaattcATGAGGACTTCCTGTCTGGTTGG |
|  | P8 | atcaccagatatctgggatccCAGATGCTGAGAATCTCACAAGCC |

Small letters indicate the homologous arm sequence (including cleavage sites of restriction enzyme and intron regions) and uppercase letters are gene-specific sequences.

Table S2. Primers for amplification of *POR* fragments

| Fragment | Primer | Sequence (5'→3') |
| --- | --- | --- |
| P1 | F1 | cttggtaccgagctcggatccATGGGAGACTCCCACGTGGA |
|  | R1 | ttggactcctCATCCAGGTTGTTCAGGGACA |
| P2 | F2 | aacctggatgAGGAGTCCAACAAGAAGCACCC |
|  | R2 | acctccttgcCCTGCTGTCGCAGCCAGG |
| P3 | F3 | cgacagcaggGCAAGGAGGTGGGGGAGA |
|  | R3 | tgctggatatctgcagaattcTACAGAAAACAGAACTTTATTCCAAGG |

Note: Lowercase letters indicate *Bam*HI (in F1) and *Eco*RI (in R3) restriction sites and linker sequences; uppercase letters are gene-specific sequences.


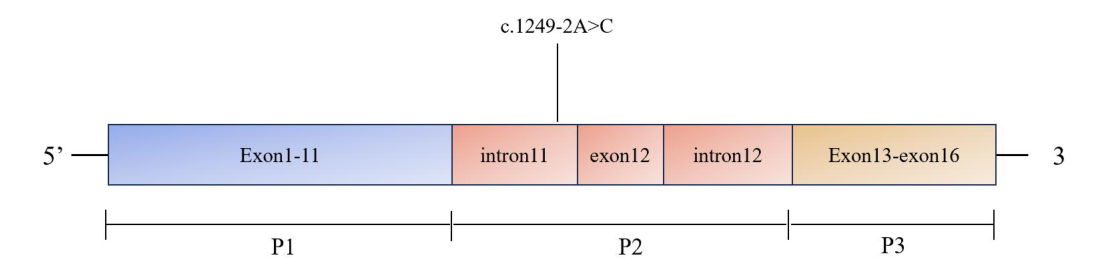


Figure S1. Structural diagram of the inserted segment
